# Evaluation of Surgical Clipping and Endovascular Coiling on Oculomotor Nerve Palsy Caused by Internal Carotid Artery Aneurysm

**DOI:** 10.3389/fneur.2020.609003

**Published:** 2020-12-11

**Authors:** Zhenqing Sun, Xueqiang Yan, Xiaolong Li, Jie Wu

**Affiliations:** Department of Neurosurgery, Guangdong 999 Brain Hospital, Guangzhou, China

**Keywords:** clipping, coiling, nerve palsy, aneurysm 2, recovery

## Abstract

**Objectives:** Internal carotid artery (ICA) aneurysm often leads to oculomotor nerve palsy (ONP) that impairs eye movement. Currently, microsurgical clipping and endovascular coiling are the two major options to treat ONP. The purpose of the current study is to compare the clinical outcomes of the two methods in patients with ONP caused by ICA aneurysm.

**Patients and Methods:** In the present study, we assessed the prognostic factors and recovery outcomes of a total of 90 ICA aneurysm-induced ONP patients, where 50 of them were treated with microsurgical clipping and 40 of them were treated with endovascular coiling. Within the endovascular coiling group, 20 of the patients were treated with balloon-assisted coiling and the other 20 were treated with stent-assisted coiling.

**Results:** Overall, we achieved a 59% (53 out of 90) full recovery rate. Both surgical clipping and endovascular coiling treatment methods achieved similar recovery outcomes in the tested patients. However, within the endovascular coiling group, balloon-assisted coiling treatment demonstrated a significantly higher full recovery rate (17 out of 20) compared to stent-assisted coiling treatment (eight out of 20).

**Conclusion:** In general, no significant difference was identified between the surgical and coiling treatments, and both procedures were considered as beneficial for ICA aneurysm-induced ONP.

## Introduction

The oculomotor nerve is also known as the third cranial nerve. It passes through the superior orbital fissure to enter the orbit, where it innervates the extrinsic eye muscle to enable the majority of eye movements. It also innervates the intrinsic eye muscle to facilitate pupillary constriction. Oculomotor nerve palsy (ONP) arises from oculomotor nerve damage and often leads to impaired eye movement and compromised pupillary reflex. Aneurysm of the blood vessels that supply the brain and the eye is one of the major causes of ONP, as a result of direct cranial nerve compression from the aneurysm (1). The internal carotid artery (ICA) is one of these vessels and is located on the inner side of the neck. Anatomically, the communicating terminal segment of ICA branches from the anterior choroidal artery and posterior communicating artery.

The prevalence of intracranial aneurysms is ~4% of the population (2). Among all types of intracranial aneurysms, the saccular type accounts for 90% of the disease; and within this type of intracranial aneurysm, ICA is the second most frequent location (2). ICA aneurysm family is quite extensive and represents serious difficulties for the surgeon due to bony obstacles and difficulty in proximal control (3, 4). One of the clinical manifestations of ICA aneurysm is ONP, as a result of the impending rupture of the aneurysm (5, 6). According to a previous population-based study, the incidence of ONP caused by third nerve palsy is ~6% (7). However, the most suitable treatment option is unknown. Many studies support microsurgical clipping as a method of directly decompressing the oculomotor nerve, whereas other studies favor endovascular coiling, which obstructs the blood supply to the aneurysm by filling up the structure with a pack of platinum coils via a micro-catheter (8–18). Balloon-assisted and stent-assisted coiling are also some of the most often used devices for endovascular coiling, with strengths and drawbacks (19, 20).

In the present study, we performed a retrospective study on 90 patients diagnosed with ICA aneurysm-induced ONP who were treated with either of the two methods to directly compare the treatment outcome.

## Materials and Methods

The study was carried out in accordance with the Code of Ethics of the World Medical Association (Declaration of Helsinki). The study protocol were approved by the local institutional review boards of the Guangdong 999 Brain Hospital (2015-09756). We obtained verbal consent from each patient at the time of diagnosis, confirming that they were willing to take part in the study. Treatment strategy was decided by taking into account the individual risks and benefits of both procedures. As a result, 50 patients received microsurgical clipping treatment and 40 patients received endovascular coiling treatment.

We performed this retrospective study on 90 ICA aneurysm patients diagnosed with ONP between 2015 and 2020. The ICA aneurysm diagnosis and location were carefully confirmed by computed tomography (CT) scan and magnetic resonance imaging (MRI). Magnetic resonance angiography (MRA) or computed tomography angiography (CTA) were not used. Complete ONP was diagnosed by the presence of all the following symptoms: ptosis (drooping of the eyelid), ophthalmoplegia (weakness or paralysis of extraocular muscle), diplopia (double vision), mydriasis (pupil dilation), and disappearance of pupillary light reflex (21). Ophthalmological examinations at diagnosis and during follow-up were performed by professional ophthalmologists. The resolution of ONP was also determined in these examinations by professional ophthalmologists. Partial ONP was diagnosed if the patient only exhibited some of the above-mentioned symptoms. A full recovery from ONP was defined as the complete elimination of all the symptoms exhibited during diagnosis. If any symptom remained by the end of the follow-up period, a partial recovery was recorded. The follow-up period was 6 months.

Microsurgical clipping was carried out via the standard pterional procedure under general anesthesia. Sylvian fissure was dissected to fully expose the aneurysm neck of the ICA for a complete clipping. When the diameter of the aneurysm exceeded a certain limit, it was pierced after the clipping to reduce its mass, as well as to verify a complete clipping of the tissue. Endovascular balloon-assisted coiling (BAC) and stent-assisted coiling (SAC) were executed under general anesthesia in all cases. The procedure was carried out following the method described previously (22). All patients who underwent endovascular coiling received antiplatelet premedication for at least 5 days. Aneurysm embolization was achieved by delivering the coil-containing micro-catheter to the tissue via the femoral artery. No striking complications were observed in any patients after the treatment. No flow-diversions were applied for any of the patients.

### Statistical Analysis

The SPSS 16.0 software (IBM Corp, Armonk, New York) was used for statistical analysis. The measurement or count data were presented as mean ± standard deviation (*SD*) and range. Normally, distributed continuous variables were compared with the independent-sample *t*-test, and non-normally distributed continuous variables were compared with the rank sum test. Multivariate Cox logistic regression analysis was used to identify the factors associated with treatment outcome. *P* < 0.05 indicated statistical significance.

## Results

### Patient Characteristics and Recovery Status

A total of 90 ICA patients were included in the present study. Of these, 50 underwent surgical clipping treatment and 40 underwent endovascular coiling treatment. Treatment methods were selected based on the general standard for cerebral aneurysm, taking into account the risks and benefits of both procedures. No prior history of diabetes or atherosclerosis was diagnosed in any of the patients. For the diagnosis of atherosclerosis, patients were first assessed by a physical exam to check for a weak or absent pulse. X-ray and/or CT scans were subsequently performed to check for signs of heart failure and hardened or narrowed arteries, respectively. The presence of an aneurysm was confirmed by digital subtraction angiography (DSA) for all patients. The degree of occlusion was assessed right after the treatment by at least two neuroradiologists using the Raymond–Roy Occlusion Classification (23). All patients showed either complete obliteration (class 1) or residual neck (class 2). The degree of aneurysm closure was assessed by DSA at 6 months after the initial treatment. The reversibility of all patients was classified as either showing improvement or no change as previously described (24) and based on the opinion of at least two neuroradiologists. The location of the ICA aneurysms was found either on the internal carotid-posterior communicating artery or the internal carotid-anterior choroidal artery for all patients (Supplementary Table 1). The contact extent between the aneurysm and the oculomotor nerve was monitored by MRI.

Detailed patient characteristics were listed in Supplementary Table 1. No differences were observed between the patients in the surgical clipping and endovascular coiling groups in terms of age, sex, body weight, body mass index (BMI), the time between ONP onset and treatment, ONP type, aneurysm diameter, location of the aneurysm, recovery status and time to full recovery (Table 1). All patients demonstrated dramatically improved oculomotor nerve function after both treatments and achieved full or partial recovery after 6 months of post-surgical follow-ups. In patients that achieved partial recovery, ptosis was the most common remaining symptom (Table 2). It is worth noting that the mortality rate was zero during the 6-months follow-up period.

**Table 1:** Comparison of the characteristics between the surgical and coiling groups.

**Characteristics**	**Surgical Clipping group (*n* = 50)**	**Endovascular coiling group (*n* = 40)**	***P*-value**
Age	62.86+/−11.95	60.35+/−12.64	0.3405
Sex (male:female)	31:19	31:19	
Body weight (kg)	56.3+/−6.3	58.5+/−5.9	0.0938
BMI	20.1+/−3.5	21.4+/−2.7	0.0693
Time between ONP onset and treatment (day)	17.96+/−8.69	18.03+/−7.88	0.9708
ONP type (complete:incomplete)	23:27	20:20	0.6439
Aneurysm diameter (mm)	8.87+/−3.19	8.09+/−3.59	0.2815
Location of aneurysm (IC-PCom: IC-Ach)	41:9	32:8	
Recovery status (full:partial)	28:22	25:15	0.1021
Time to full recovery (day)	115.54+/−46.58	105.3+/−41.57	0.2802

*IC-Pcom, internal carotid-posterior communicating; IC-Ach, internal carotid-anterior choroidal; BMI, body mass index*.

**Table 2:** Remaining symptoms in partially recovered patients of the surgical and coiling groups.

**Remaining symptoms in partially recovered patients**	**Surgical clipping group (*n* = 22)**	**Endovascular coiling group (*n* = 15)**
Ptosis	11	8
Ophthalmoplegia	5	3
Diplopia	6	4
Mydriasis	4	4
Disappearance of pupillary light reflex	5	4

We further compared the characteristics of the patients within the endovascular coiling group, operated by either balloon-assisted coiling or stent-assisted coiling. We found that significantly more patients achieved full recovery upon balloon-assisted coiling treatment (17 out of 20) compared to patients treated with stent-assisted coiling (eight out of 20) (Table 3).

**Table 3:** Comparison of the characteristics between the BAC and SAC groups.

**Characteristics**	**BAC group (*n* = 20)**	**SAC group (*n* = 20)**	***P*-value**
Age	60.3+/−13.11	60.4+/−12.5	0.9804
Sex (male:female)	10:10	11:9	
Body weight (kg)	57.2+/−5.1	58.3+/−5	0.4913
BMI	20.9+/−3.3	21.8+/−1.9	0.3266
Time between ONP onset and treatment (day)	18.75+/−7.59	17.3+/−8.29	0.5672
ONP type (complete:incomplete)	10:10	10:10	
Aneurysm diameter (mm)	8.53+/−3.95	765+/−3.23	0.448
Recovery status (full:partial)	17:3	8:12	0.0102
Time to full recovery (day)	110.8+/−39.83	99.8+/−43.56	0.4097

*BAC, balloon-assisted coiling; SAC, stent-assisted coiling*.

### Multivariate Analysis

To identify the factors relating to post-operative recovery status, we performed a stratified statistical analysis by adding factors of aneurysm diameter, the time between ONP onset and treatment, preoperative complete ONP rate, and treatment method into a multivariate Cox logistic regression analysis. The analytical method was adopted with corrections based on age and sex. Surgical clipping and endovascular coiling treatment methods had a similar impact on the postoperative recovery rate of both the completely recovered and partially recovered patients after the correction for age and sex (Table 4). We also applied the same analytical model to compare the factors between the balloon-assisted and stent-assisted coiling groups and found that the balloon-assisted coiling method yielded a significantly better recovery outcome (Table 5).

**Table 4:** Multivariate Cox Logistic analysis of factors relating to postoperative recovery in surgical and coiling groups.

**Factors**	**HR (95% CI)**	***P*-value**
Age	1.205 (0.887–1.341)	0.2341
Sex	0.932 (0.658–1.097)	0.2433
Aneurysm diameter	1.154 (0.785–1.267)	0.5447
Time between ONP onset and treatment	1.087 (0.765–1.233)	0.3464
Preoperative complete ONP	1.389 (0.987–1.467)	0.3889
Treatment method	5.456 (3.289–7.765)	0.4955

**Table 5:** Multivariate Cox Logistic analysis of factors relating to postoperative recovery in BAC and SAC groups.

**Factors**	**HR (95% CI)**	***P*-value**
Age	1.342 (0.897–1.503)	0.3469
Sex	1.138 (0.987–1.302)	0.3752
Aneurysm diameter	1.567 (1.112–1.754)	0.2145
Time between ONP onset and treatment	1.743 (1.245–1.902)	0.3212
Preoperative complete ONP	0.976 (0.712–1.168)	0.2344
Treatment method	7.878 (5.643–9.321)	<0.001

*BAC, balloon-assisted coiling; SAC, stent-assisted coiling*.

## Discussion

Surgical clipping focuses on controlling the mass effect of the aneurysm to the oculomotor nerve (25). It was the only treatment option for aneurysm bleeding before the 1990's. Endovascular treatment strategy became an option for ONP in the late 1990's, which targeted the pulsation stress to the oculomotor nerve without affecting the aneurysmal mass (9). Since then, a long debate has been ongoing in the clinical field to figure out which is the better treatment option for ONP. Several studies have clearly stated that microsurgical clipping provides better functional outcomes for the treatment of ONP aneurysms and should be considered as the first-choice (12, 13, 15). Conversely, a number of other studies have reported no significant difference in the functional outcomes of ONP recovery between the two treatments (8, 10, 16, 17). The comparison outcome of all previous reports is summarized in Table 6. Our present study generally agrees with the second pile of studies showing no obvious differences between the two treatment options in terms of the extent and length of recovery. Furthermore, our results suggest that the balloon-assisted coiling method might have a better impact on achieving full ONP recovery status.

**Table 6:** Summary of the comparison outcome of all previous reports.

**References**	**Type of ONP**	**No. of patients treated by SC**	**No. of patients treated by EC**	**Recovery outcome comparison**
Ahn et al. (8)	PComA aneurysm-induced	7	10	No statistical difference was observed in outcome between SC and EC.
Brigui et al. (10)	PComA aneurysm-induced	7	13	Both SC and EC were safe and effective methods in term of functional recovery
Chen et al. (12)	PComA aneurysm-induced	7	6	SC was associated with a higher probability of complete recovery from ONP than EC.
Guresir et al. (13)	PComA aneurysm-induced	4	7	A systemic review suggested that a significantly higher portion of patients were resolved after SC compared with EC.
Khan et al. (15)	PComA aneurysm-induced	8	9	Complete recovery is more commonly associated with SC than with EC
Mino et al. (16)	ICA aneurysm-induced	9	8	Both SC and EC were beneficial for achieving functional recovery of ONP
Nam et al. (17)	UIA aneurysm-induced	8	6	No significant differences were observed in the clinical outcome between SC and EC.
Patel et al. (14)	PComA aneurysm-induced	9	9	No significant difference was found between SC and EC.
Tan et al. (18)	PComA aneurysm-induced	132	43	SC may be more advantageous than EC
Birchall et al. (9)	PComA aneurysm-induced	0	3	Assessed EC treatment only
Chalouhi et al. (11)	PComA aneurysm-induced	0	37	Assessed EC treatment only

*PComA, posterior communicating artery; ICA, internal carotid artery; UIA, unruptured intracranial aneurysm; SC, surgical clipping; EC, endovascular coiling*.

From a technical point of view, microsurgical clipping not only reduces the aneurysmal mass, it also eliminates the pulsation stress on the oculomotor nerve by directly collapsing the aneurysm; whereas endovascular coiling only reduces the pulsation stress in the aneurysm without affecting its mass. The dual effects of microsurgical clipping might account for its preference by some of the doctors. However, endovascular coiling does not involve any incision on the skull, which minimizes the risk of the treatment procedure as compared to microsurgical clipping and might make it a better option for elderly patients (26).

The full recovery rate in the present study is 59% (53 out of 90). It falls within the reported full recovery rate range of the two treatments, which is between 30 and 86% after surgical clipping and between 0 and 100% after endovascular coiling (1, 6, 9, 10, 27). The big variance in the recovery rate is likely a result of different standards at different institutions. Our study not only adds to current statistics but also suggests that the BAC method might have a better recovery outcome compared to the SAC method. This outcome is not in line with a previous study that compares BAC and SAC treatment methods on unruptured ICA aneurysms, where they reveal both methods are safe and effective techniques with no differences in morbi-mortality and recurrence rates between the two groups (28). Both balloon and stent are known to be the representative assistant devices for the treatment of ICA aneurysms because ICA is the largest intracranial artery and therefore is more accessible for navigation (29, 30). Since we only have 20 patients for each group in the present study as compared to a total of 190 patients in the previous study (28), we are not confident enough to deduce any underlying reasons to account for such difference before more similar studies with bigger patient population come out. In addition, there is a difference between the imaging follow-up methods of the two studies, where the previous study used MRA (28) while we used DSA. This also might account for the discrepancies in the outcome of the two studies. This study proposes a hypothesis that BAC might be a better treatment option for ICA aneurysm as compared to SAC.

There are certain limitations associated with the current study. First, because the treatment method was selected by the patients following the clinical team's suggestion, it was not possible to achieve randomization in this study. Second, a larger patient population size would further strengthen these conclusions. Third, the patient population analyzed is heterogeneous, including patients with aneurysms at different locations and different durations of symptoms before treatment. Fourth, the follow-up period in this study is relatively short. Further large scale prospective comparative studies will be needed to overcome these limitations.

## Conclusion

In summary, this study had a similar satisfying full recovery rate for ONP in both microsurgical clipping and endovascular coiling groups, suggesting that both methods are equally effective against ONP. Within the endovascular coiling group, the balloon-assisted coiling method achieves a better recovery outcome compared to the stent-assisted method. However, further investigations with larger patient populations are needed to confirm this observation.

## Data Availability Statement

The original contributions presented in the study are included in the article/Supplementary Material, further inquiries can be directed to the corresponding author/s.

## Ethics Statement

The studies involving human participants were reviewed and approved by institutional review boards of the Guangdong 999 Brain Hospital. The ethics committee waived the requirement of written informed consent for participation.

## Author Contributions

ZS, XY, and XL collected, analyzed, and interpreted the data. JW designed the study and wrote the manuscript. All authors read and approved the final manuscript.

## Conflict of Interest

The authors declare that the research was conducted in the absence of any commercial or financial relationships that could be construed as a potential conflict of interest.

## Abbreviations

ICA: Internal carotid artery
ONP: oculomotor nerve palsy
MRI: magnetic resonance imaging
BAC: balloon-assisted coiling
SAC: stent-assisted coiling.
