# Supplementary material for: Evaluation of Surgical Clipping and Endovascular Coiling on Oculomotor Nerve Palsy Caused by Internal Carotid Artery Aneurysm
Source: Front Neurol. 2020 Dec 11;11:609003. doi: 10.3389/fneur.2020.609003 (PMC7759633; doi:10.3389/fneur.2020.609003)
Supplement: Supplementary file 1 [file Table_1.DOCX]

**Table S1**: Patient characteristics. CO, complete ONP; PO, partial ONP; FR, full recovery; PR, partial recovery; SC, surgical clipping; EC endovascular coiling; BAC, balloon-assisted coiling; SAC, stent-assisted coiling; IC-Pcom, internal carotid-posterior communicating; IC-Ach: internal carotid-anterior choroidal.

| Patient No. | Age | Sex | time between ONP onset and treatment (day) | ONP type | Location of aneurysm | Aneurysm diameter (mm) | Recovery status | time to full recovery (day) | Treatment group |
| --- | --- | --- | --- | --- | --- | --- | --- | --- | --- |
| 1 | 71 | M | 30 | CO | IC-PCom | 11.5 | FR | 131 | SC |
| 2 | 55 | M | 7 | CO | IC-PCom | 12.8 | FR | 83 | SC |
| 3 | 54 | M | 15 | CO | IC-PCom | 6.0 | FR | 56 | SC |
| 4 | 45 | M | 21 | CO | IC-PCom | 13.3 | PR | 89 | SC |
| 5 | 72 | M | 7 | PO | IC-PCom | 7.9 | FR | 153 | SC |
| 6 | 79 | M | 5 | CO | IC-PCom | 10.9 | PR | 175 | SC |
| 7 | 71 | M | 22 | PO | IC-Ach | 7.8 | FR | 168 | SC |
| 8 | 66 | M | 9 | PO | IC-PCom | 10.9 | PR | 30 | SC |
| 9 | 51 | M | 23 | CO | IC-Ach | 5.5 | FR | 177 | SC |
| 10 | 66 | M | 22 | CO | IC-Ach | 6.6 | FR | 134 | SC |
| 11 | 44 | M | 6 | CO | IC-PCom | 7.1 | FR | 173 | SC |
| 12 | 76 | M | 22 | CO | IC-PCom | 5.0 | PR | 177 | SC |
| 13 | 78 | M | 28 | PO | IC-PCom | 12.7 | FR | 109 | SC |
| 14 | 59 | M | 24 | CO | IC-Ach | 11.5 | FR | 143 | SC |
| 15 | 81 | M | 6 | PO | IC-PCom | 7.9 | FR | 173 | SC |
| 16 | 71 | M | 20 | CO | IC-Ach | 10.2 | FR | 164 | SC |
| 17 | 51 | M | 14 | CO | IC-PCom | 12.2 | FR | 73 | SC |
| 18 | 74 | M | 5 | CO | IC-Ach | 13.9 | FR | 135 | SC |
| 19 | 60 | M | 21 | CO | IC-PCom | 4.8 | FR | 49 | SC |
| 20 | 53 | M | 8 | CO | IC-Ach | 12.2 | FR | 35 | SC |
| 21 | 70 | M | 13 | PO | IC-PCom | 5.4 | FR | 136 | SC |
| 22 | 55 | M | 28 | PO | IC-PCom | 8.3 | FR | 105 | SC |
| 23 | 66 | M | 28 | CO | IC-PCom | 5.3 | FR | 98 | SC |
| 24 | 42 | M | 30 | CO | IC-PCom | 10.8 | PR | 142 | SC |
| 25 | 40 | M | 25 | CO | IC-PCom | 13.0 | FR | 122 | SC |
| 26 | 68 | M | 28 | CO | IC-PCom | 6.5 | FR | 161 | SC |
| 27 | 46 | M | 5 | CO | IC-PCom | 13.7 | FR | 178 | SC |
| 28 | 48 | M | 29 | CO | IC-PCom | 6.3 | FR | 111 | SC |
| 29 | 65 | M | 17 | CO | IC-PCom | 10.4 | FR | 88 | SC |
| 30 | 61 | M | 13 | CO | IC-PCom | 6.6 | FR | 108 | SC |
| 31 | 69 | F | 10 | PO | IC-Ach | 7.4 | PR | 162 | SC |
| 32 | 76 | F | 14 | PO | IC-PCom | 13.1 | PR | 60 | SC |
| 33 | 79 | F | 29 | PO | IC-PCom | 3.5 | PR | 58 | SC |
| 34 | 66 | F | 30 | PO | IC-PCom | 5.1 | PR | 47 | SC |
| 35 | 68 | F | 30 | PO | IC-PCom | 5.1 | PR | 131 | SC |
| 36 | 67 | F | 13 | PO | IC-PCom | 10.9 | PR | 176 | SC |
| 37 | 58 | F | 23 | PO | IC-PCom | 7.0 | PR | 48 | SC |
| 38 | 43 | F | 17 | PO | IC-PCom | 4.9 | PR | 36 | SC |
| 39 | 70 | F | 14 | PO | IC-PCom | 7.8 | PR | 123 | SC |
| 40 | 77 | F | 25 | PO | IC-PCom | 3.6 | PR | 53 | SC |
| 41 | 76 | F | 25 | PO | IC-PCom | 12.4 | PR | 157 | SC |
| 42 | 52 | F | 10 | PO | IC-PCom | 7.5 | PR | 143 | SC |
| 43 | 63 | F | 30 | PO | IC-PCom | 11.3 | PR | 64 | SC |
| 44 | 80 | F | 20 | PO | IC-PCom | 9.8 | FR | 98 | SC |
| 45 | 79 | F | 13 | PO | IC-PCom | 5.1 | FR | 159 | SC |
| 46 | 41 | F | 9 | PO | IC-PCom | 7.0 | FR | 142 | SC |
| 47 | 56 | F | 5 | PO | IC-PCom | 12.8 | PR | 137 | SC |
| 48 | 52 | F | 30 | PO | IC-PCom | 14.0 | PR | 42 | SC |
| 49 | 71 | F | 9 | PO | IC-Ach | 12.1 | PR | 141 | SC |
| 50 | 62 | F | 11 | PO | IC-Ach | 6.3 | PR | 124 | SC |
| 51 | 57 | F | 22 | PO | IC-PCom | 13.4 | PR | 110 | EC (BAC) |
| 52 | 76 | F | 26 | PO | IC-PCom | 10.7 | PR | 135 | EC (BAC) |
| 53 | 43 | F | 24 | PO | IC-PCom | 3.2 | PR | 40 | EC (BAC) |
| 54 | 50 | F | 24 | PO | IC-PCom | 10.8 | PR | 94 | EC (BAC) |
| 55 | 56 | F | 16 | PO | IC-PCom | 12.0 | PR | 80 | EC (BAC) |
| 56 | 42 | F | 26 | CO | IC-PCom | 3.0 | FR | 140 | EC (BAC) |
| 57 | 42 | F | 5 | CO | IC-PCom | 13.6 | FR | 95 | EC (BAC) |
| 58 | 78 | F | 23 | CO | IC-PCom | 13.2 | FR | 96 | EC (BAC) |
| 59 | 49 | F | 7 | CO | IC-PCom | 4.3 | FR | 165 | EC (BAC) |
| 60 | 64 | F | 20 | CO | IC-PCom | 7.4 | FR | 147 | EC (BAC) |
| 61 | 53 | M | 7 | PO | IC-PCom | 11.2 | FR | 53 | EC (BAC) |
| 62 | 71 | M | 19 | CO | IC-PCom | 12.2 | FR | 153 | EC (BAC) |
| 63 | 85 | M | 17 | CO | IC-PCom | 6.8 | FR | 124 | EC (BAC) |
| 64 | 59 | M | 29 | CO | IC-PCom | 3.1 | PR | 66 | EC (BAC) |
| 65 | 71 | M | 27 | CO | IC-Ach | 3.2 | FR | 100 | EC (BAC) |
| 66 | 78 | M | 10 | CO | IC-Ach | 9.8 | FR | 144 | EC (BAC) |
| 67 | 49 | M | 27 | CO | IC-PCom | 3.2 | FR | 68 | EC (BAC) |
| 68 | 66 | M | 10 | CO | IC-PCom | 11.5 | FR | 158 | EC (BAC) |
| 69 | 52 | M | 22 | PO | IC-PCom | 10.4 | FR | 173 | EC (BAC) |
| 70 | 65 | M | 14 | CO | IC-PCom | 7.5 | PR | 75 | EC (BAC) |
| 71 | 70 | M | 16 | PO | IC-PCom | 13.0 | PR | 37 | EC (SAC) |
| 72 | 85 | M | 13 | CO | IC-PCom | 8.0 | FR | 117 | EC (SAC) |
| 73 | 71 | M | 30 | CO | IC-PCom | 3.1 | FR | 70 | EC (SAC) |
| 74 | 52 | M | 11 | CO | IC-Ach | 7.7 | FR | 77 | EC (SAC) |
| 75 | 42 | M | 11 | CO | IC-Ach | 3.2 | FR | 162 | EC (SAC) |
| 76 | 58 | M | 27 | CO | IC-Ach | 10.2 | PR | 101 | EC (SAC) |
| 77 | 70 | M | 30 | CO | IC-PCom | 12.2 | PR | 162 | EC (SAC) |
| 78 | 52 | M | 9 | CO | IC-PCom | 4.9 | PR | 80 | EC (SAC) |
| 79 | 76 | M | 21 | PO | IC-PCom | 9.9 | FR | 62 | EC (SAC) |
| 80 | 62 | M | 24 | PO | IC-PCom | 9.2 | FR | 81 | EC (SAC) |
| 81 | 73 | M | 6 | PO | IC-PCom | 3.4 | PR | 119 | EC (SAC) |
| 82 | 47 | F | 30 | PO | IC-PCom | 5.5 | PR | 45 | EC (SAC) |
| 83 | 54 | F | 22 | PO | IC-PCom | 7.8 | PR | 64 | EC (SAC) |
| 84 | 64 | F | 15 | PO | IC-PCom | 8.6 | FR | 132 | EC (SAC) |
| 85 | 51 | F | 25 | PO | IC-PCom | 6.7 | FR | 137 | EC (SAC) |
| 86 | 54 | F | 8 | PO | IC-PCom | 3.3 | FR | 179 | EC (SAC) |
| 87 | 71 | F | 6 | PO | IC-PCom | 7.0 | PR | 128 | EC (SAC) |
| 88 | 44 | F | 17 | PO | IC-Ach | 10.2 | FR | 70 | EC (SAC) |
| 89 | 42 | F | 9 | PO | IC-Ach | 5.7 | FR | 36 | EC (SAC) |
| 90 | 70 | F | 16 | PO | IC-Ach | 13.4 | FR | 137 | EC (SAC) |
